# Newborn screening for congenital adrenal hyperplasia in Tokyo, Japan from 1989 to 2013: a retrospective population-based study

**DOI:** 10.1186/s12887-015-0529-y

**Published:** 2015-12-15

**Authors:** Atsumi Tsuji, Kaoru Konishi, Satomi Hasegawa, Akira Anazawa, Toshikazu Onishi, Makoto Ono, Tomohiro Morio, Teruo Kitagawa, Kenichi Kashimada

**Affiliations:** Department of Pediatrics and Developmental Biology, Tokyo Medical and Dental University, Tokyo, Japan; Tokyo Health Service Association, Newborn Screening, Tokyo, Japan; Kinki Central Hospital, Hyogo, Japan

**Keywords:** Congenital adrenal hyperplasia, Newborn screening, 21-hydroxylase deficiency

## Abstract

**Background:**

Congenital adrenal hyperplasia (CAH) cause life-threatening adrenal crisis. It also affects fetal sex development and can result in incorrect sex assignment at birth. In 1989, a newborn screening program for congenital adrenal hyperplasia (CAH) was introduced in Tokyo. Here we present the results of this screening program in order to clarify the efficiency of CAH screening and the incidence of CAH in Japan.

**Method:**

From 1989 to 2013, a total of 2,105,108 infants were screened for CAH. The cutoff level for diagnosis of CAH was adjusted for gestational age and birth weight.

**Results:**

A total of 410 infants were judged positive, and of these, 106 patients were diagnosed with CAH, indicating a positive predictive value (PPV) of 25.8 %. Of the 106 patients, 94 (88.7 %) were diagnosed with 21-OHD. Of these 94 patients, 73 were diagnosed with the salt wasting form, 14 with the simple virilising form and 7 with the nonclassical form (NC21OHD). The mean birth weight and gestational age were 3192 ± 385 g and 38.9 ± 1.38 weeks. 11 out of 44 female patients were assigned as female according to their screening result.

**Conclusions:**

These data suggest that the newborn screening in Tokyo was effective, especially for sex assignment and preventing fatal adrenal crisis. The incidence of CAH was similar to that measured in previous Japanese screening studies, and it was also similar to that of western countries. The incidence of NC21OHD in Japan in the present study was lower than that in western countries as previous studies reported. The screening program achieved higher PPV than previous CAH screening studies, which might be due to the use of variable cutoffs according to gestational age and birth weight. However, most of the neonates born at 37 weeks or less that were referred to hospital were false-positives. Further changes are needed to reduce the number of false positive preterm neonates.

## Background

Congenital adrenal hyperplasia (CAH) is an inherited disorder caused by the loss or severely impaired activity of steroidogenic enzymes involved in cortisol biosynthesis. More than 90 % of cases result from 21-hydroxylase deficiency (21-OHD) caused by mutations in *CYP21A2* [1, 2]. The prevalence of 21-OHD has been estimated at 1 in 18,000. According to the clinical phenotypes, the disease is classified into three forms, the salt wasting (SW) form and the simple virilising (SV) form, which are also called the classical form, and the nonclassical (NC) form. The SW form is the severest. Virilisation of external genitalia in newborn females and precocious puberty due to overproduction of androgens from the adrenal cortex are major clinical problems of both the SW and SV forms. In the SW form, in addition to overproduction of androgens, aldosterone is deficient and it causes life-threatening adrenal crisis.

In order to prevent life-threatening adrenal crisis and to help make the appropriate sex assignments in affected female patients, newborn mass screening programs for CAH have been introduced in many countries including Japan [3–5]. The aim of our study was to summarize the results of the past 23 years of newborn mass screening for CAH in Tokyo. Specifically, we wished to determine the efficiency of CAH screening and the incidence of CAH in Tokyo.

This study is the largest retrospective analysis of CAH newborn screening by using a single screening program in East Asia [6–10]. Tokyo is the largest city accounting for more than 10 % of the population in Japan [11], and to date, more than two million neonates have been screened. False positives for CAH in preterm infants is one of the major concerns of newborn screening programs [2]. In a pilot study from 1984 to 1987, we found that we could reduce the number of false positives by using higher cut-offs for preterm or low birth weight infants from that for term infants, and used these different criteria throughout the screening program.

The positive predictive value of our study was higher than those of previous reports of CAH screenings.

## Methods

### Subjects

From 1 January, 1989 to 31 March, 2013, neonates born in Tokyo were screened. Basically we recommended collecting the blood sample from the age of 4 to 7 days, and clinical data was obtained by follow-up survey from each hospital where neonates judged as positive at screening were referred.

### Measurement of 17-OHP and criteria

**Fig. 1:**
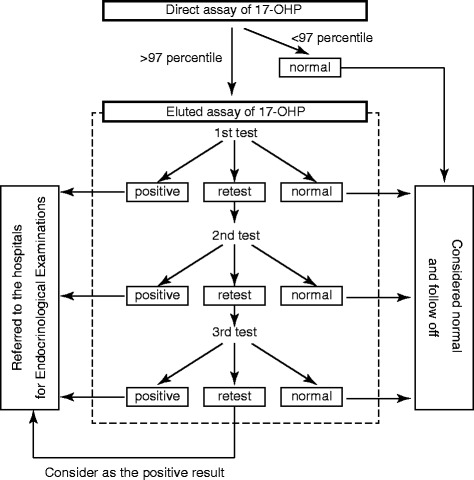
Algorithm of CAH screening in Tokyo. Abbreviation: 17-OHP: 17-hydroxyprogesterone

Blood samples were collected by a heel prick blotted on a filter paper after written informed consent was obtained from parents. The level of 17-hydroxyprogesterone (17-OHP) was initially determined by enzyme linked immunosorbent assay (ELISA) (Siemens Medical Solutions Diagnostics, CA, U.S.) without steroid extraction. Blood samples in the 97th percentile or higher of 17-OHP values were subjected to the second ELISA (Eiken Chemical CO., LTD, Tokyo, Japan) after steroid extraction (Fig. 1). The measured values on the second assay were doubled to be equivalent to the serum levels. Sex, birth weight, and gestational age were recorded in the application form for the screening test, so we obtained these data from all neonates who underwent the screening. The cutoff level of 17-OHP was adjusted according to 1) gestational age (GA) at birth, 2) corrected gestational age at the time of the test and 3) body weight at the time of the test (Table 1). The cutoffs were determined according to our pilot study of serum 17-OHP levels in term and preterm infants. The criteria for preterm and low birth weight infants were used from the start of the screening in Tokyo. The algorithm and criteria of the screening are shown in Table 1 and Fig. 1. Briefly, the patients whose results were “re-tests” were recalled to repeat a test of 17-OHP measurement, and the test was performed at the hospital where the patients were born. If the level of 17-OHP was higher than 60 nmol/L or still higher than normal range on the third test, the patient was considered to be positive. The patients with “positive” results were referred to pediatric endocrinologists for further endocrinological evaluation.

**Table 1:** Criteria of CAH mass screening in Tokyo

<Criteria according to the gestational age >					
Gestational age at birth (weeks)^a^		≤29	30–34	35–36	37–
Corrected gestational age (weeks)^b^		≤31	32–35	36–37	38–
<Criteria according to weight >^c,d^					
Body weight (g)		≤999	1,000–1,999	2,000–2,499	2,500–
Cutoff level of 17-OHP [nmol/L]	Retest^e^	60	45	24	15
	Positive^f^		60	60	60

^a^Samples collected before the age of 7 days

^b^Samples collected at the age of 7 days or after

^c^1^st^ test: Body weight = Birth weight. 2^nd^ test and after: Body weight = Corrected body weight calculated by the formula as below. Corrected body weight at test (g) = birth weight (g) + (age at test – 7) × 20 (g)

^d^For infants born small or large for gestational age, either the criteria of gestational age (corrected gestational age) or body weight was applied, whichever was lower value

^e^recall for the second (or the third) test of the screening

^f^refer to hospitals for further endocrinological examinations

### Follow-up survey

We performed follow-up survey of the patients who were referred to hospitals. We collected clinical information of the patients from the physicians of the hospitals. The collected information included the diagnosis of the patients including the type of CAH, laboratory data before the start of the treatment (17-OHP, Na, K), and the brief clinical course during the early infantile period. We gathered the surveys of all patients who were referred to the hospitals. The present retrospective analysis was approved by the ethics committee of Tokyo Health Service Association (No. 2014–2–1).

## Results

Firstly, we comprehensively analysed our data, including the incidence and the positive predictive value (PPV) of the screening. Subsequently, we examined the clinical details of the CAH patients who were identified by our screening, and finally, one of the purposes of the screening, sex assignment issue, was analysed.

### Incidence and positive predictive value of the screening

A total of 2,105,108 neonates were screened. Coverage of the screening was 93 % of newborn babies in Tokyo registered in Vital Statics of Japan [12]. Of these, 410 neonates had positive results and were referred to hospitals. The median age at first screening was 5 days (range 0–62 days), consistent with our recommendation. Of the 410 neonates, 106 were diagnosed with CAH, resulting in an incidence of 1:19,859 (Table 2). Diagnosis of CAH was based on the endocrinological data and physical findings [13]. Genetic tests were not carried out in all cases.

**Table 2:** Positive predictive value of the screening and incidence of CAH in Tokyo. Overview of the screening results

Category	Number (Percent)
Total infants tested	2,105,108 (100)
Retested	7,940 (0.38)
Positive	410 (0.02)
CAH patients	106 (0.005)
Positive predictive value	25.8 %
Incidence of CAH by screening	1:19,859

Of 300 infants born at term, 100 were diagnosed as having CAH, resulting in a positive predictive value (PPV) of full-term neonates of 33.3 %. Even though the criteria were applied according to gestational age, 99 (24.1 %) were preterm infants with a positive result. Thus, the PPV of preterm neonates who were born before 37 weeks gestation was only 2 % (2/99), resulting in 25.8 % (106/410) of the total PPV of the screening (Table 3).

**Table 3:** Positive predictive value of the screening and incidence of CAH in Tokyo. Positive predictive value on term and preterm infants

	Total	Term	Preterm	Data not available
			(<37 weeks)	
	Number of infants			
Infants with positive result	410	300	99	11
CAH patients	106	100	2	4
Positive predictive value	25.8 %	33.3 %	2.0 %	

**Fig. 2:**
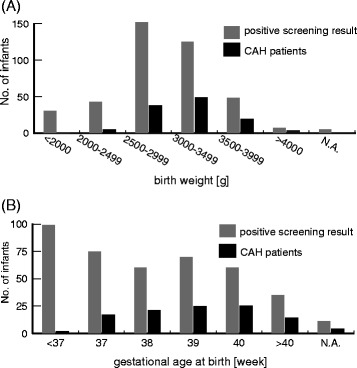
Birth weights (**a**) and gestational ages (**b**) of patients and newborns judged as positive in CAH screening. Abbreviation: CAH: congenital adrenal hyperplasia; N.A.: data not available

The gestational age of the 21-OHD patients was distributed in a bell-shape curve with a single peak (Fig. 2), however, the gestational age distribution in the referred neonates showed two peaks at 39 and 37 weeks, resulting in lower predictive value of the screening for infants born at 37 weeks gestation or before. These data suggest that neonates, even at 37 weeks of gestational age, tend to show unspecific elevation of serum levels of 17-OHP by cross-reaction for adrenal steroids from fetal adrenal cortex (Fig. 2).

### Clinical details of CAH patients identified by the screening

The gestational ages and the birth weights were 38.9 ± 1.38 weeks and 3192 ± 385 g (Table 4). In 2009, the average birth weight of single births in Japan was 3020 g, and the incidence of preterm births was 4.7 % [14], which are not significantly different from those of the 21-OHD patients in Tokyo.

**Table 4:** Clinical characteristics and the details of the screening of 106 CAH patients. Characteristics of 106 CAH patients

	Number		Percent
Sex			
Male	56		(52.8)
Female	44		(41.5)
Changed from male to female		2	
Assigned to female by screening		9	
Data not available	6		
Gestational Age			
Preterm (<37 weeks)	2		(1.90)
Term	100		(94.3)
Data not available	4		
Form of CAH			
21-OHD	94		(88.7)
Salt Wasting (SW)		73	
Simple Virilising (SV)		14	
Nonclassical (NC)		7	
3β-HSDD	2		(1.89)
Data not available	10		
Gestational Age [weeks] (Mean ± SD)	38.9 ± 1.38		
Birth weight [g] (Mean ± SD)	3192 ± 385		

Two preterm neonates were diagnosed with 21-OHD. Both were born at 36 weeks and their birth weights were 2570 g and 2770 g, respectively. None of the 21-OHD patients were born before 36 weeks. Only one patient was born as low birth weight infant with 2380 g at 40 weeks.

Information on the type of CAH was available for 96 patients in the survey. All but two of these patients had 21-OHD. In addition, two of these patients had 3β-hydroxysteroid dehydrogenase deficiency. The most frequent type of 21-OHD was the salt wasting form, accounting for 73 of the 94 patients. Fourteen of the 21-OHD patients had the simple virilising form and seven had the nonclassical forms. The incidence of nonclassical forms was low, approximately 1:300,729 as reported previously in Japan [15–17].

**Fig. 3:**
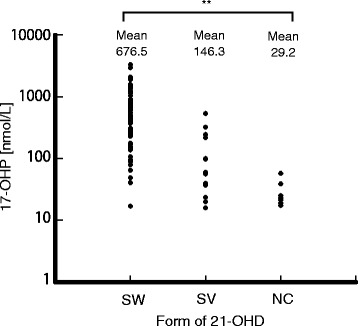
Serum levels of 17-OHP in CAH patients at the first tests. Abbreviation: 17-OHP: 17-hydroxyprogesterone; 21-OHD: 21-hydroxylase deficiency; SW: salt wasting; SV: simple virilising; NC: nonclassical. **, *p < 0.01*(ANOVA)

The mean values of the levels of 17-OHP on the first test in SW, SV and NC were 676.5, 146.3, and 29.2 nmol/L, respectively (Fig. 3). Although these values were significantly different, they substantially overlapped, suggesting that it is inappropriate to predict the form of CAH according to the value of 17-OHP.

On the first test, most SW patients (94.5 %) showed remarkably elevated levels of 17-OHP, and were referred to hospitals (Table 5). While, four SW patients (Nos. 53, 84, 99, 101) showed mildly elevated levels of 17-OHP on the first test (Table 6) and required repeated tests. These results suggest that mildly elevated 17-OHP does not exclude the possibility of classical 21-OHD. On the other hand, none of the NC patients were discovered on the first test, suggesting that it is not likely to be the NC form of 21-OHD (NC21OHD) whose 17-OHP was remarkably elevated on the first test (Table 5). No fatal cases were reported by follow-up survey.

**Table 5:** Clinical characteristics and the details of the screening of 106 CAH patients. The number of tests to be assessed positive in each form of 21-OHD

Number of test	SW	SV	NC	Total
	(*n* = 73)	(*n* = 14)	(*n* = 7)	(*n* = 94)
	Number of patients (%)			
1	69 (94.5)	8 (57.1)	0 (0)	77 (81.9)
2	3 (4.11)	3 (21.4)	2 (28.5)	8 (8.51)
≥3	1 (1.37)	3 (21.4)	5 (71.4)	9 (9.57)

Upper: The number of the patients

Lower (%): The proportion to the total number of patients in each form

**Table 6:** Clinical characteristics and the details of the screening of 106 CAH patients. 17-OHP values of SW patients tested repeatedly

	17-OHP result [nmol/L]		
	First test	Second test	Third test
Twice			
Patient No.53	49.09	339.97	
Patient No.99	16.97	90.90	
Patient No.101	40.60	244.22	
Three times			
Patient No.84	47.57	27.27	23.94

### Screening-assisted sex assignments

Of the 106 CAH patients, 56 were males, 44 were females and the information of the sex in 6 cases was not available on the survey (Table 4). Two of the patients originally thought to be males were reassigned as females according to the screening results. Nine patients were assigned as females according to the screening results (Table 4).

If the patients without information of assigned sex were female with ambiguous genitalia, the total number of female patients might be 50, and the sex assignment of 17 female patients would have been assisted by the screening results.

## Discussion

Our study revealed the incidence of CAH in Tokyo was 1/19,859. In Japan, newborn screening has been carried out in each prefecture independently with different criteria and different follow-up survey systems. Thus, it has been difficult to have a large-scale study of the screening. Suwa’s meta-analysis in Japan and Morikawa’s analysis in Sapporo reported the incidence of CAH was 1/18,827 and 1/20,756 [10, 18]. The incidences in these studies were very similar to our data. We assume that the incidence of CAH in Japan is approximately 1/20,000.

Our data suggests that the screening was performed properly. One of the aims of the screening is to assist proper sex assignment in 46, XX patients. It was reported that, before the neonatal screening program started, 12 % of 46,XX patients were incorrectly assigned to male [19]. Therefore, our data strongly suggest that the screening assisted in the sex assignment of CAH patients.

The another objective of the screening is to prevent fatal adrenal crisis during the neonatal period. The screening program might contribute to decreasing the mortality by preventing neonatal fatal adrenal crisis with few false negative cases. Despite our screening program lacked the system to detect false negative patients, none of the cases who were missed by the screening program were reported to be fatal by pediatric endocrinologists in Tokyo. Additionally, no childhood deaths in recent years in Japan have been attributed to CAH [20]. Further, no mortalities from CAH have been attributed to false negatives after the start of newborn screening programs in Japan [21]. The screening programs have decreased mortality rate due to CAH from 6.8 % to 1.2–4.0 % [21].

Because CAH screening results in many false positives in preterm infants [2], we used cut-off criteria for preterm infants and low birth weight infants that were higher than those used for term infants. The recall ratio (0.19 %) was lower and the PPV (25.8 %) was higher than those of other reports (Table 7) [5, 9, 10, 22–25], especially when compared to two other studies from Japan that did not use different cut-off criteria for preterm infants. Indeed, the ratio of the number of referred term infants to the number of preterm infants (3.03) was much higher than the ratios in other reports (Table 7), suggesting that our program eliminated false positive cases of preterm or low birth weight infants. We concluded that using cut-off criteria for preterm infants and low birth weight infants was effective at reducing false positive cases.

**Table 7:** Proportion of preterm infants among published studies

		Number of Patients Referred to Clinical Hospital						PPV, %	Variable 17-OHP cutoff criteria	
Reference	n	Total (%)		Term	Preterm	N.A.	A/B		GA	Birth weight
				(A)	(B)					
The Netherland, 2001 [24]	87,827	224	(0.255)	70	150	4	0.47	5.9	Yes	Yes
France, 2012 [22]	6,012,798	15,407	(0.256)	1,058	10,562	3,787	0.10	2.3	No	No
Sweden, 2014 [5]	2,737,932	1728	(0.063)	874	854	0	1.02	13.4	Yes	Yes
Niigata, Japan, 2011 [9]	478,337	242	(0.050)	69	173	0	0.39	10.7	No	No
Sapporo, Japan, 2014 [10]	251,922	880	(0.349)	170	708	2	0.24	N.A.	No	No
Tokyo, Japan, 2015	2,105,108	410	(0.019)	300	99	11	3.03	25.8	Yes	Yes

Abbreviation: PPV: Positive Predictive Value; GA: Gestational Age; N.A.: data not available

Even though our PPV was higher than the PPVs in other screening systems, it was still only 25 %, indicating that the efficiency of our screen program at eliminating false positive cases is limited. Unspecific cross-reactions for adrenal steroids from fetal adrenal cortex have been reported to cause false positive results in preterm infants [26]. The high false-positive rate is one of the major concerns of CAH newborn screening, and introducing novel assay systems with higher specificity for 17-OHP might achieve more efficient screening [27–29]. A recently developed assay system, that uses tandem mass spectrometry, has been reported to have extremely high specificity for steroid assays and might be considered for a future assay system [30, 31].

The incidence of NC21OHD patients identified by the screening was lower than the incidence in European countries and the U.S. [4, 25], and is consistent with previous reports from Japan [15–17]. It is difficult to predict the incidence of NC21OHD according to the newborn screening results because patients with the NC form are usually missed by newborn screening [32]. However, it has been assumed that the incidence of NC21OHD in Japan would be lower than that of western countries [16, 17]. In western countries, nonclassical cases are mainly caused by V281L mutation in *CYP21A2* that is rare in Japanese patients [1, 16, 33]. In Japan, a P30L mutation is the major genetic cause for NC21OHD, although the frequency of P30L in Japan is much lower than that of V281L in western countries, resulting in a lower incidence of NC21OHD in Japan [16].

In our study, two patients were diagnosed with 3β-HSDD, suggesting that a careful diagnostic approach is essential to differentiate other types of CAH from 21-OHD. The serum level of 17-OHP is known to be elevated in other forms of CAH, such as 11β-hydroxylase deficiency (11-OHD) and cytochrome P450 oxidoreductase deficiency (PORD). Even in 3β-HSDD, the 17-OHP level is reported to be paradoxically elevated, and occasionally similar to that of 21-OHD [34]. These diagnostic problems are potential clinical pitfalls in diagnosing the type of CAH. Including sex assignment, a different clinical approach is required for each type of CAH. In terms of the type of CAH, it should be noted that 11-OHD patients were not reported in our screening survey. The incidence of 11-OHD has been reported to be much higher than that of 3β-HSDD, and we cannot exclude the possibility that some 11-OHD patients were incorrectly diagnosed.

The limitation of this study is lack of the system to collect the information on false negative cases and didn’t detect precise number of false negatives.

## Conclusion

Newborn screening in Tokyo was performed effectively for sex assignment and preventing fatal adrenal crisis, contributing to correct sex assignment and reduce mortality. The incidence of classical 21-OHD was similar to that of western countries, although the incidence of NC was much lower than reported previously in western countries. The PPV appeared to be improved by adjusting the cutoffs for gestational age and for body weight, although the PPV for preterm infants was still low. To reduce the number of false positive cases, assay systems with higher specificity are needed.

## Abbreviations

CAH: congenital adrenal hyperplasia
17-OHP: 17-hydroxyprogesterone
21-OHD: 21-hydroxylase deficiency
3β-HSDD: 3β-hydroxysteroid dehydrogenase deficiency
11-OHD: 11β-hydroxylase deficiency
PORD: cytochrome P450 oxidoreductase deficiency
PPV: positive predictive value
SW: salt wasting
SV: simple virilising
NC: nonclassical
NC21OHD: nonclassical form of 21-OHD
GA: gestational age
